# Influence of urbanization on schistosomiasis infection risk in Anhui Province based on sixteen year's longitudinal surveillance data: a spatio-temporal modelling study

**DOI:** 10.1186/s40249-023-01163-3

**Published:** 2023-11-29

**Authors:** Xin Liu, Yang Sun, Yun Yin, Xiaofeng Dai, Robert Bergquist, Fenghua Gao, Rui Liu, Jie Liu, Fuju Wang, Xiao Lv, Zhijie Zhang

**Affiliations:** 1 College of Geodesy and Geomatics, Shandong University of Science and Technology, Qingdao, Shandong China; 2 No. 8 Institute of Geology and Mineral Resources Exploration of Shandong Province, Rizhao, Shandong China; 3 School of Public Health, Fudan University, Shanghai, China; 4 Key Laboratory of Public Health Safety, Ministry of Education, Shanghai, China; 5 Ingerod, Brastad, Sweden; 6 Anhui Institute of Schistosomiasis Control, Hefei, Anhui China

**Keywords:** Urbanization, Night-time light data, Schistosomiasis, Spatio-temporal analysis, Geographically and temporally weighted regression

## Abstract

**Background:**

Urbanization greatly affects the natural and social environment of human existence and may have a multifactoral impact on parasitic diseases. Schistosomiasis, a common parasitic disease transmitted by the snail *Oncomelania hupensis*, is mainly found in areas with population aggregations along rivers and lakes where snails live. Previous studies have suggested that factors related to urbanization may influence the infection risk of schistosomiasis, but this association remains unclear. This study aimed to analyse the effect of urbanization on schistosomiasis infection risk from a spatial and temporal perspective in the endemic areas along the Yangtze River Basin in China.

**Methods:**

County-level schistosomiasis surveillance data and natural environmental factor data covering the whole Anhui Province were collected. The urbanization level was characterized based on night-time light data from the Defense Meteorological Satellite Program Operational Linescan System (DMSP-OLS) and the National Polar-Orbiting Partnership's Visible Infrared Imaging Radiometer Suite (NPP-VIIRS). The geographically and temporally weighted regression model (GTWR) was used to quantify the influence of urbanization on schistosomiasis infection risk with the other potential risk factors controlled. The regression coefficient of urbanization was tested for significance (α = 0.05), and the influence of urbanization on schistosomiasis infection risk was analysed over time and across space based on significant regression coefficients. Variables studied included climate, soil, vegetation, hydrology and topography.

**Results:**

The mean regression coefficient for urbanization (0.167) is second only to the leached soil area (0.300), which shows that the urbanization is the most important influence factors for schistosomiasis infection risk besides leached soil area. The other important variables are distance to the nearest water source (0.165), mean minimum temperature (0.130), broadleaf forest area (0.105), amount of precipitation (0.073), surface temperature (0.066), soil bulk density (0.037) and grassland area (0.031). The influence of urbanization on schistosomiasis infection risk showed a decreasing trend year by year. During the study period, the significant coefficient of urbanization level increased from − 0.205 to − 0.131.

**Conclusions:**

The influence of urbanization on schistosomiasis infection has spatio-temporal heterogeneous. The urbanization does reduce the risk of schistosomiasis infection to some extend, but the strength of this influence decreases with increasing urbanization. Additionally, the effect of urbanization on schistosomiasis infection risk was greater than previous reported natural environmental factors. This study provides scientific basis for understanding the influence of urbanization on schistosomiasis, and also provides the feasible research methods for other similar studies to answer the issue about the impact of urbanization on disease risk.

**Supplementary Information:**

The online version contains supplementary material available at 10.1186/s40249-023-01163-3.

## Background

As one of the most important human activities, urbanization has a great impact on people's lives [1, 2]. In 1950, the global urban population accounted for only 30% of the total population but by 2018 this proportion had risen to 55%, with the global urbanized population exceeding that of the rural population [2]. Over the past half century, China has undergone urbanization at an unprecedented speed, with the proportion of the urban population increasing from 10.6% in 1949 to 63.9% in 2020 [3]. Urbanization may have multiple effects on the presence of parasitic diseases. In addition, dense human population and high-frequency contacts expand the spread of diseases [4] and when municipal services are deficient, polluted water and soil often result in stronger breeding of intermediate hosts and vectors. On the other hand, cities may bring better health in terms of basic living standards that help blocking disease transmission, in particular parasitic infections [5, 6]. More adequate medical resources are also conducive to the detection and treatment of patients. In addition, environmental changes of climate and landscapes brought by urbanization have effects on the survival of animal hosts, animal vectors and pathogens, which we still do not know [7–9].

Schistosomiasis is a parasitic disease caused by the trematode worm *Schistosoma* that seriously endangers people's health and safety. As the second largest parasitic disease in the world after malaria, it is prevalent in 78 tropical and subtropical countries and regions [10, 11]. In China, the disease is zoonotic and relies on one single intermediate snail host *Oncomelania hupensis,* an amphibious snail that spreads in water-logged areas. Schistosomiasis was once been prevalent in 12 provinces/autonomous regions/municipalities and remains a threat along the Yangtze River. The endemic areas are densely populated and the susceptible population base remains large in spite of long-term integrated strategies to control schistosomiasis in epidemic areas [12, 13], such as replacing cattle with machines, housing livestock in barns, chemical control of snails, and ensuring the provision of safer water and adequate sanitation. As of 2021, there were still 451 schistosomiasis-endemic counties, 27,571 endemic villages, totalling an at-risk population of 73,250,600, and a snail-inhabited area of 1911.6 km^2^ [14].

Previous studies on factors influencing the distribution of schistosomiasis have found that meteorological factors (temperature, humidity, etc.), climate change [15, 16] and micro-environmental conditions (vegetation cover, soil composition, water environment, etc.) [17] have significant effects on the survival of snails and the spread of schistosomiasis. In addition, social factors, such as demographic and cultural characteristics [18], economic levels [19] and sanitation facilities [20, 21] may also play a role in the transmission. While these studies have individually explored the influence of natural or social factor, a comprehensive study on the impact of urbanization on schistosomiasis, considering both natural and social factors remains lacking. China is currently undergoing significant transformations characterized by agricultural mechanization, urbanization of land use and population movement. The continuous improvement of urban infrastructure and living conditions has profoundly changed the social environment. Rapidly increasing population mobility, with urban population size and density, has led to changes in the ecological environment. However, the impact of changes in social and natural factors in the process of urbanization with respect to schistosomiasis infection risk and the mechanism of action have not been discussed. This issue is important as it is integral to our comprehension of the overall situation and holds the potential to enhance public health security and promote sustainable development of urbanization.

Based on 16 years of continuous surveillance of schistosomiasis data in Anhui Province, this study used a geographically and temporally weighted regression (GTWR) model to evaluate the dynamic impact of urbanization on schistosomiasis with aim of revealing the spatio-temporal characteristics of the impact of urbanization on schistosomiasis infection risk. We expect that our approach will provide a reference basis for further improvement of schistosomiasis control in the province and offer valuable insights for the continued healthy development of urbanization in China.

## Methods

### Study area

**Fig. 1:**
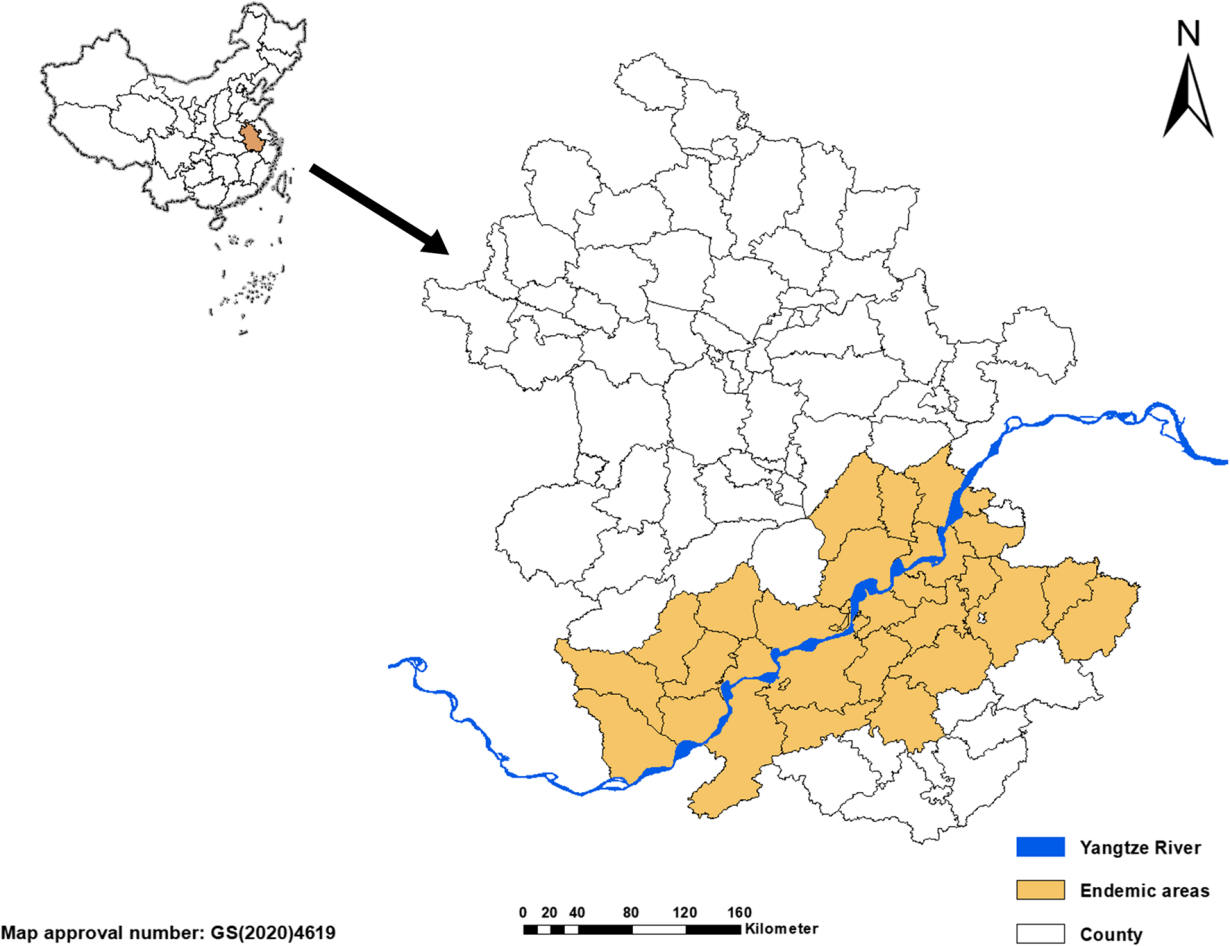
The endemic area of *Schistosomiasis japonica* in Anhui Province, People's Republic of China

Anhui Province is located in a subtropical monsoon climate zone in east China along the rivers of Yangtze and Huai He, and close to the Yangtze River Delta. Most of the land is flat covering an area of 139,600 km^2^ with a population of about 63,659 million (2019). It has jurisdiction over 16 prefecture-level cities and 105 county-level administrative units, more geographical details see Fig. 1.

### Schistosomiasis infection rate data

This study used schistosomiasis infection data at the county level that were obtained from annual cross-sectional surveys carried out by health professionals of Anhui Institute of Parasitic Disease (AIPD) in 29 counties between 2000 and 2015. There are no new local cases of schistosomiasis in Anhui Province after 2015 [22, 23], so the study period was confined to before 2015. The number of schistosomiasis cases are generally determined by the two-pronged diagnostic approach [24–26] based on indirect haemagglutination serology followed by a confirmatory Kato-Katz parasitological, faecal test [27]. Testing included all residents 5 to 65 years old in epidemic villages and the infection rate was calculated by the number of blood test participants and the number of those with positive faecal test results as follows [28]:

1 $$\begin{array}{c}{\text{Schistosomiasis}} \, {\text{infection}} \, {\text{rate}} = \frac{{\text{number of faecal positive test participants}}} {{\text{number of blood test participants}}}\end{array}$$

### Data source

We used night-time light data to indicate the urbanization level of Anhui Province. These data were collected from the US Defense Meteorological Satellite Program–Operational Linescan System (DMSP–OLS) with 1-km spatial resolution, 1-year temporal resolution and a 6-bit radiation resolution, and the National Polar–Orbiting Partnership’s Visible Infrared Imaging Radiometer Suite (NPP–VIIRS) with 500-m spatial resolution, 1-month temporal resolution and 12-bit radiation resolution, both available from the US National Oceanic and Atmospheric Administration (NOAA) (http://ngdc.noaa.gov/eog/download.html). The DMSP-OLS data ranged from 2000 to 2013, and the NPP-VIIRS data ranged from 2012 to 2015. So, the combination of the two kinds of night-time light data was needed to describe the urbanization level from 2000 to 2015.

The geographic distribution of schistosomiasis is generally consistent with the geographic distribution of its sole snail intermediate host, and the factors affecting this distribution thus affect the distribution of the disease itself. Natural factors such as climate, soil, hydrology, vegetation and topography influence the survival, growth and reproduction of snails [29–35] as well as the infection risk of schistosomiasis, so information on these factors was collected. We used 42 indicators in 6 categories (Table 1) [36].

**Table 1:** Data on environmental factors affecting schistosomiasis distribution

Category	Serial number	Data	Spatial scale	Time horizon	Time scale	Data source
Climate	1	Average temperature	1 km	2000–2015	Month	National Meteorological Information Center (http://data.cma.cn)
	2	Mean minimum temperature	1 km	2000–2015	Month	
	3	Mean maximum temperature	1 km	2000–2015	Month	
	4	Extreme minimum temperature	1 km	2000–2015	Month	
	5	Extreme maximum temperature	1 km	2000–2015	Month	
	6	Amount of precipitation	1 km	2000–2015	Month	
	7	Daily precipitation > 0.1 mm	1 km	2000–2015	Month	
	8	Average relative humidity	1 km	2000–2015	Month	
	9	Sunshine duration	1 km	2000–2015	Month	
	10	Surface temperature	1 km	2000–2015	Month	LAADS DAAC (http://ladsweb.nascom.nasa.gov/data/search.html)
Soil	11	Leached soil area	1 km	1995	Year	Resource and Environment Science and Data Center (http://www.resdc.cn)
	12	Semi-aqueous soil area	1 km	1995	Year	
	13	Artificial soil area	1 km	1995	Year	
	14	Iron bauxite soil area	1 km	1995	Year	
	15	Sand content	1 km	1995	Year	
	16	Silt content	1 km	1995	Year	
	17	The clay content	1 km	1995	Year	
	18	Soil bulk density	30 s	2013	Year	National Tibetan Plateau Data Center (http://westdc.westgis.ac.cn)
	19	Soil porosity	30 s	2013	Year	NASA (https://disc.gsfc.nasa.gov/datasets?keywords=GLDAS)
	20	Soil organic matter content	30 s	2013	Year	
	21	Soil potassium content	30 s	2013	Year	
	22	Soil nitrogen content	30 s	2013	Year	
	23	Soil phosphorus content	30 s	2013	Year	
	24	Soil PH	30 s	2013	Year	
	25	Soil moisture	0.25 degrees	2000–2015	Month	
	26	Vegetation coverage	1 km	2000–2015	Month	LAADS DAAC (http://ladsweb.nascom.nasa.gov/data/search.html)
Vegetation	27	Coniferous forest area	1 km	2001	Year	Resource and Environment Science and Data Center (http://www.resdc.cn)
	28	Broadleaf forest area	1 km	2001	Year	Resource and Environment Science and Data Center (http://www.resdc.cn)
	29	Brush area	1 km	2001	Year	
	30	Grass area	1 km	2001	Year	
	31	Cultivated vegetation area	1 km	2001	Year	
	32	Nearest water source distance	90 m	2015	Year	
Hydrology	33	Water area	1 km	2015	Year	
	34	Elevation	90 m	2003	Year	
Landform	35	Plain area	1 km	2009	Year	
	36	Platform area	1 km	2009	Year	
	37	Hilly area	1 km	2009	Year	
	38	Mountain area	1 km	2009	Year	
	39	Cultivated land area	1 km	2015	Year	
Land use	40	Woodland area	1 km	2015	Year	
	41	Grassland area	1 km	2015	Year	
	42	Construction land area	1 km	2015	Year	

NASA: United States National Aeronautics and Space Administration; LAADS DAAC: Level-1 and Atmosphere Archive & Distribution System–Distributed Active Archive Center; Sec and degree are grid sizes of datasets used

### Data pre-processing

Unlike DMSP-OLS that had noise removed, there was a considerable amount of background noise in NPP-VIIRS night-time light data. Therefore, we first extracted the pixel value that had non-zero in DMSP-OLS night-time light data as the mask and then overlay it to NPP-VIIRS data, with the area outside the mask taken as the noise part. Finally, we extracted the NPP-VIIRS night-time light data from the mask. The maximum night-time light intensity in the urban centre was selected as the maximum threshold and the light intensity beyond this threshold in the non-urban centre areas was assigned as the maximum threshold of the urban central areas [37]. After that, two kinds of night-time light data were clipped using the administrative boundary data of Anhui Province obtained from the National Geomatics Center of China.

The average night-time light index (ANLI) [38] for each county was the average of the pixel brightness values within each geographic space. In order to obtain ANLIs from 2000 to 2015, we had to integrate these two data with different temporal scales and different periods of time. Since the DMSP-OLS data is given by year and the NPP-VIIRS by month, we used the averaging method to convert the monthly data of NPP-VIIRS to the annual average index. Curve fitting was performed with the ANLIs of NPP-VIIRS and DMSP-OLS at the county level in Anhui in 2012 and 2013. With the NPP-VIIRS index as the independent variable and the DMSP-OLS index as the dependent one, the fitting equation was a cubic polynomial: $y=0.018{x}^{3}-0.677{x}^{2}+9.183{x}+2.750$ (*F* = 3.252, *P* < 0.001, *R*^2^ = 0.957). When applying the fitting formula, the 2014–2015 NPP-VIIRS index was converted into the DMSP-OLS index, and finally the county-level urbanization level in Anhui Province from 2000 to 2015 was obtained according to the night-time optical index.

The climate data are point data obtained from meteorological stations, so Kriging interpolation was used to interpolate the point data into surface data to obtain a 1 km × 1 km grid meteorological data. The soil surface temperature data and vegetation coverage data were obtained from Landsat image inversion (https://landsat.gsfc.nasa.gov/) to obtain 1-km raster data. Zonal statistics was used to calculate the average of grid data of each county and the temporal resolution of the data were unified to year, i.e. the average of 12-month observations. The minimum and maximum normalization was used to normalize each factor data. The software used was ArcGIS, version 10.2 (ESRI Inc., Redlands, CA, USA), ENVI, version 5.3 (Research System Inc.; Boulder, CO, USA) and Matlab (https://www.mathworks.com/products/matlab.html).

### Variable filtering

The specific steps of variable filtering in the analysis of the correlation between the schistosomiasis infection rate and other variables were the following:

Step 1: All variables with no statistically significant Pearson correlation of the schistosomiasis infection rate (test level *α* = 0.1) were excluded.

Step 2: Multicollinearity tests were conducted on the variables included in the correlation analysis using the variance inflation factor (VIF) method [39]. Variables with VIF < 10 were retained.

Step 3: Backward regression [40] was carried out to establish a regression equation for the variables having passed the collinearity test and perform significance testing on the regression coefficients (*α* = 0.1).

### Geographically and temporally weighted regression (GTWR)

The GTWR model [41, 42] is an extension of the geographically weighted regression (GWR) model [43]. Due to the spatial and temporal non-stationarity [42] in parameter estimation, spatial coordinates and temporal coordinates were added to the model to calculate the spatio-temporal weight matrix. Spatio-temporal non-stationarity means that the parameters describing spatio-temporal relationships vary in time and space. The GTWR model of urbanization and environmental factors on schistosomiasis infection rate [41, 42] was based on the formula:

2 $${{{Y}}_{{i}}}={{\beta}_{{0}}}\left( {{{u}}_{{i}}}{,}{{{v}}_{{i}}}{,}{{{t}}_{{i}}} \right){+}\underset{{k}}{\mathop \sum }\,{{\beta}_{{k}}}\left( {{{u}}_{{i}}}{,}{{{v}}_{{i}}}{,}{{{t}}_{{i}}} \right){{{X}}_{{ik}}}{+}{{\varepsilon}_{{i}}}$$

where ${{Y}}_{{i}}$ is the dependent variable of the $i^{th}$ sample point; $X_ik$ is the $k^{th}$ independent variable of the $i^{th}$ sample point; ${\varepsilon}_i$ is the random error; $u_i$ is the longitude coordinate of the $i^{th}$ sample point, $v_i$ is the latitude coordinate of the $i^{th}$ sample point; $t_i$ is the time coordinate of the $i^{th}$ sample point; $\left(u_i,v_i,t_i\right)$ is the spatio-temporal dimension coordinates of the $i^{th}$ sample point; ${\beta}_{0}\left({{u}}_{{i}}{,}{{v}}_{{i}}{,}{{t}}_{{i}}\right)$ is the constant term of the $i^{th}$ sample point; and ${\beta}_{{k}}\left({{u}}_{{i}}{,}{{v}}_{{i}}{,}{{t}}_{{i}}\right)$ is the regression coefficient of the ${{k}}^{{th}}$ independent variable at the ${{i}}^{{th}}$ sample point, with the least squares estimation [44] as follows:

3 $$\begin{array}{c}\widehat{\beta}\left({{u}}_{{i}}{,}{{v}}_{{i}}{,}{{t}}_{{i}}\right){=}{\left[{{X}}^{{T}}{{W}}\left({{u}}_{{i}}{,}{{v}}_{{i}}{,}{{t}}_{{i}}\right){{X}}\right]}^{-1}{{X}}^{{T}}{{W}}\left({{u}}_{{i}}{,}{{v}}_{{i}}{,}{{t}}_{{i}}\right){{Y}}\end{array}$$

where the spatio-temporal weight matrix $W\left({{u}}_{{i}}{,}{{v}}_{{i}}{,}{{t}}_{{i}}\right)$ is a diagonal matrix of $n \times n$; $W(u_{i} ,v_{i} ,t_{i} ) = diag(W_{i1} ,W_{i2} ,...,W_{ij} ,...,W_{in} )$; and ${{W}}_{{ij}}$ is the attenuation function of spatio-temporal distance [45], calculating spatio-temporal weight matrix by attenuation function:

4 $$\begin{array}{c}{{W}}_{{ij}}{=}{{e}}{{x}}{{p}}\left[-\frac{{\left({{d}}_{{ij}}^{{ST}}\right)}^{2}}{{{h}}^{2}}\right]\end{array}$$

where ${{h}}$ is a non-negative parameter called the spatio-temporal bandwidth, which represents the spatio-temporal range affected by the sample point.

To obtain the optimal bandwidths, the minimum second-order corrected Akaike Information Criterion (AICc) value was selected. The spatio-temporal distance ${{d}}^{{ST}}$ is calculated as:

5 $$\begin{array}{c}{{d}}^{{ST}}{=}\sqrt{{\lambda}\left[{\left({{u}}_{{i}}-{{u}}_{{j}}\right)}^{2}{+}{\left({{v}}_{{i}}-{{v}}_{{j}}\right)}^{2}\right]{+}{\mu}{\left({{t}}_{{i}}-{{t}}_{{j}}\right)}^{2}}\end{array}$$

where ${\left({{u}}_{{i}}-{{u}}_{{j}}\right)}^{2}{+}{\left({{v}}_{{i}}-{{v}}_{{j}}\right)}^{2}$ is the spatial distance between sample points ${{i}}$ and ${{j}}$ , ${{({t}_{i}-{t}_{j})}^{2}}$ is the temporal distance between sample points ${{i}}$ and ${{j}}$; and ${\lambda}$ and ${\mu}$ are the scaling factor of spatial distance and temporal distance, respectively.

Taking the infection rate of schistosomiasis as the dependent variable and the significant influencing factors which passed the variable filtering including the urbanization level as the independent ones, the GTWR model, the ordinary least squares model (OLS) [46] and the GWR model were set up [47]. AICc and the coefficient of determination (*R*^2^) were used to evaluate the goodness of fit of the above three models looking for the models with lower AICc values and higher *R*^2^ values. The degree of influence of the independent variables was ranked according to the mean value of the regression coefficients of the respective variables.

## Results

### Schistosomiasis infection rate and urbanization level

**Fig. 2:**
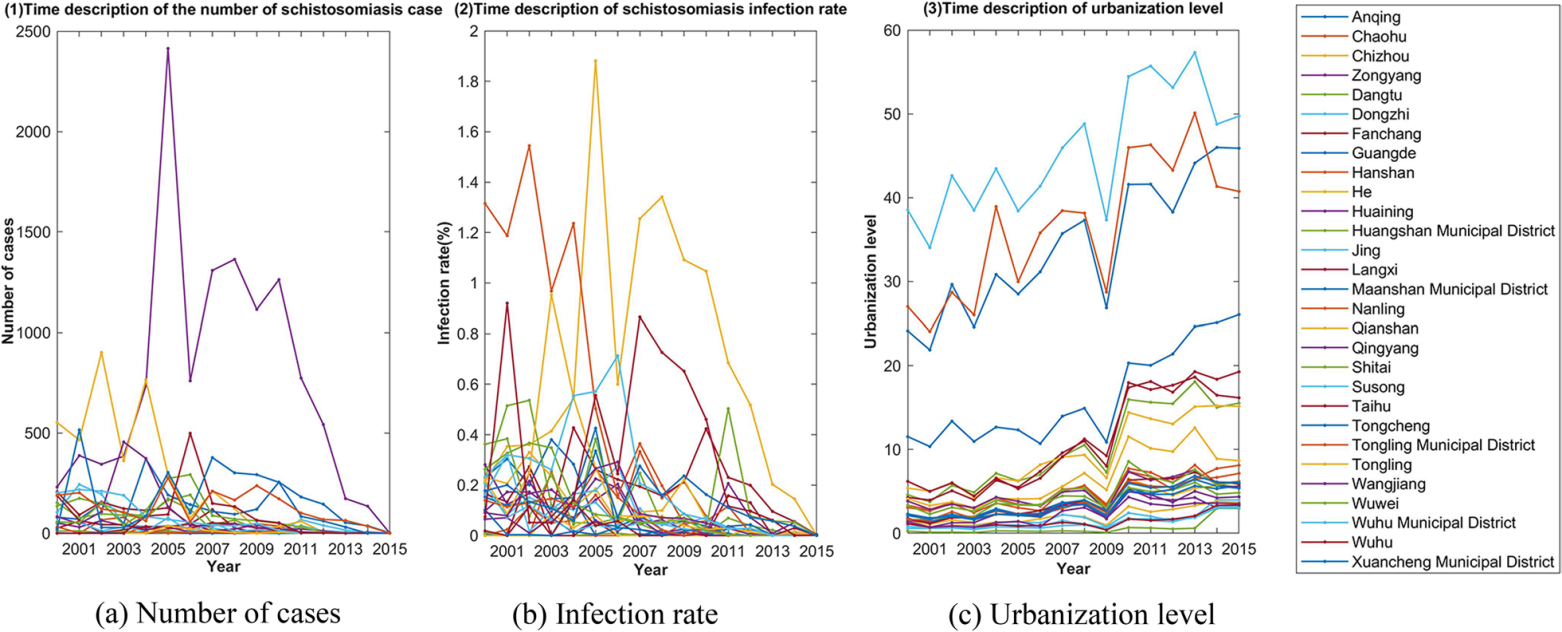
The number of cases (**a**), schistosomiasis infection rate (**b**) and urbanization level (**c**) in the endemic counties of Anhui Province

The number of cases and the infection rates of schistosomiasis in the 29 endemic counties of Anhui Province from 2000 to 2015 are shown in Fig. 2a and b. The rates fluctuated from 2000 to 2006, decreased after 2007 and tended towards zero at the end of the study period. Between 2000 and 2009, the urbanization level was in a state of fluctuation but has been in an overall upward trend since then as shown in Fig. 2c.

### GTWR model analysis

Eight natural factors were retained through the variable filtering: leached soil area, distance to nearest water source, surface temperature, soil bulk density, broadleaf forest area, grassland area, amount of precipitation and mean minimum temperature (Additional file 1). The accuracy of fitting the schistosomiasis infection rate by OLS, GWR and GTWR models is shown in Additional file 2. It was found that the AICc value of GTWR model was the smallest and its *R*^2^ was the largest. The results showed that the GTWR model was superior compared to the other two models.

The statistics of the urbanization level and other influence factors in the GTWR model are shown in Table 2. The regression coefficients involved in the statistics in the table are the standardized regression coefficients; the larger the absolute value of the standardized regression coefficients, the greater their influence on schistosomiasis infection rate. The minimum, maximum and quartiles reflect the variation in the regression coefficients of the variables across regions, and it as a response to the change in the degree of influence of each variable. It was found that the most influential factors, those with the average regression coefficient > 0.1, were leached soil area (0.300), the urbanization level (0.167), the distance to the nearest water source (0.165), mean minimum temperature (0.130), an broadleaf forest area (0.105).

**Table 2:** Statistics of regression coefficients of influencing factors of schistosomiasis in GTWR model

Variable	Mean	Minimum	Maximum	First quartile	Median	Third quartile
Leached soil area (km^2^)	0.300	0.015	0.349	0.293	0.307	0.320
Urbanization level	0.167	0.004	0.225	0.131	0.175	0.201
Nearest water source (dist.) (m)	0.165	0.010	0.196	0.144	0.172	0.186
Mean minimum temperature (℃)	0.130	0.118	0.142	0.125	0.130	0.134
Broadleaf forest area (km^2^)	0.105	0.077	0.118	0.099	0.105	0.110
Amount of precipitation (mm)	0.073	0.047	0.113	0.055	0.069	0.091
Surface temperature (℃)	0.066	0.031	0.078	0.063	0.066	0.070
Soil bulk density (g/cm^3^)	0.037	0.011	0.101	0.026	0.038	0.044
Grassland area (km^2^)	0.031	0.002	0.166	0.014	0.023	0.046

### Spatial distribution of urbanization influence on schistosomiasis infection risk

**Fig. 3:**
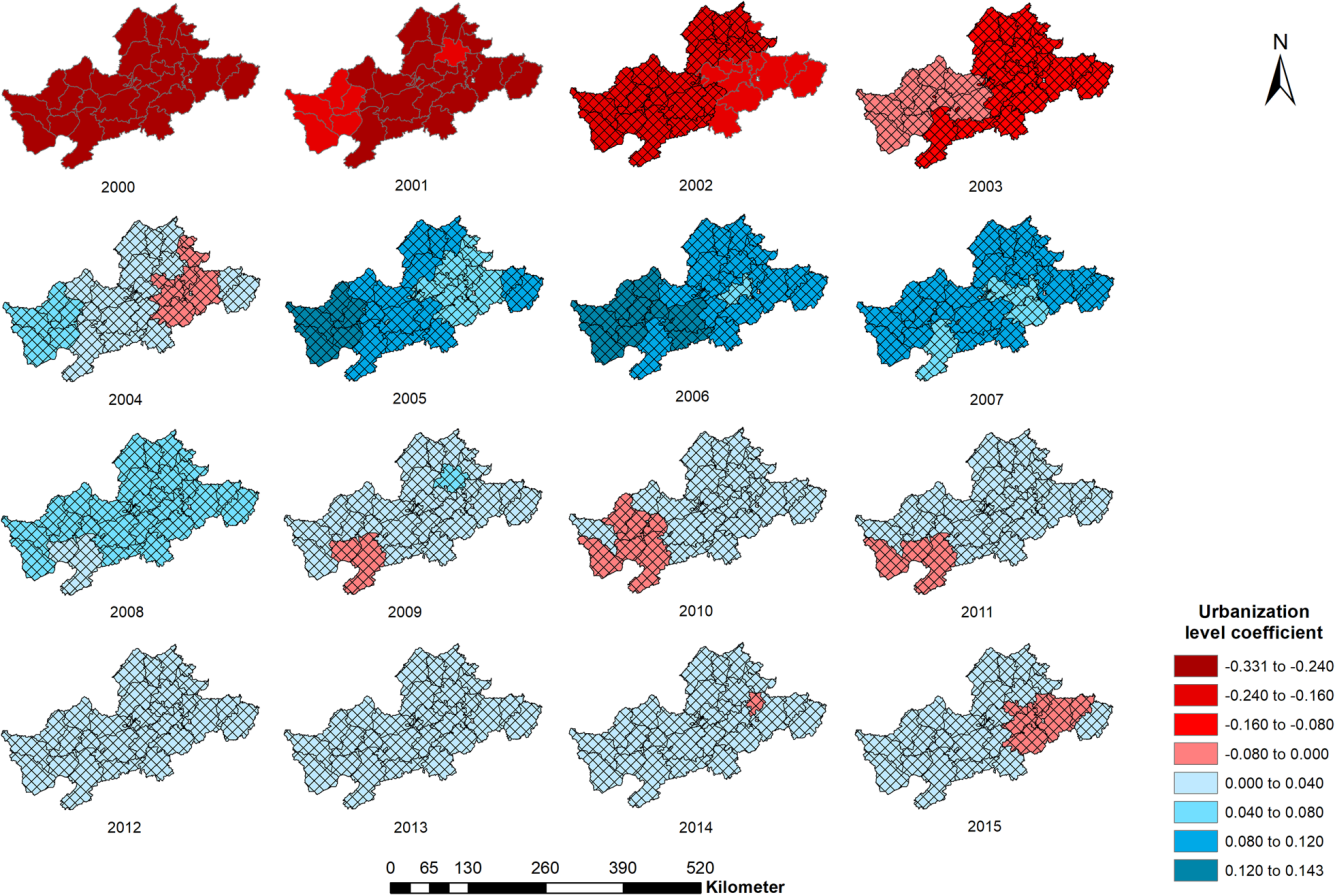
Spatial distribution of the urbanization influence coefficient on schistosomiasis 2000–2015

The spatial distribution of the urbanization influence coefficient on schistosomiasis in each county is shown in Fig. 3, which clearly reveals that the urbanization level coefficient of each county was significantly negative, and that the influence degree decreased between 2000 and 2002. After 2003, the regression coefficient of urbanization level increasingly reached the level of no statistical significance. The gray grid layer illustrates the area where the regression coefficient of the urbanization level had no statistical significance (*P* > 0.05).

### Temporal distribution of urbanization influence on schistosomiasis infection risk

The annual influence coefficient of urbanization on schistosomiasis in Anhui Province was calculated as the average coefficient of urbanization in each county every year (Table 3). It can be seen that the average urbanization influence coefficient from 2000 to 2002 was negative and that the influence degree decreased from − 0.205 to − 0.131. The impact of urbanization on schistosomiasis risk from 2003 to 2015 was, however, not statistically significant. It appeared that urbanization can decrease the spread of schistosomiasis and with the passage of time the urbanization level increases, while the influence gradually decreases (Fig. 3 and Table 3).

**Table 3:** Statistics of the influence coefficient of urbanization on schistosomiasis infection risk

Year	Mean	Minimum	Maximum	Variance
2000	− 0.205	− 0.225	− 0.193	0.0000357
2001	− 0.177	− 0.189	− 0.163	0.0000221
2002	− 0.131	− 0.142	− 0.122	0.0000221
2003	− 0.070	− 0.087	− 0.059	0.0000527
2004	− 0.004	− 0.027	0.014	0.0001256
2005	0.046	0.021	0.070	0.0001605
2006	0.062	0.041	0.080	0.0000859
2007	0.046	0.033	0.052	0.0000254
2008	0.024	0.010	0.035	0.0000351
2009	0.010	− 0.004	0.022	0.0000355
2010	0.003	− 0.008	0.012	0.0000211
2011	0.002	− 0.004	0.005	0.0000056
2012	0.003	0	0.007	0.0000025
2013	0.003	− 0.001	0.007	0.0000031
2014	0.002	− 0.002	0.006	0.0000031
2015	0.001	− 0.003	0.005	0.0000029

## Discussion

The purpose of this study was to analyse the impact of urbanization on schistosomiasis infection risk in Anhui Province from 2000 to 2015. The GTWR model has been established to analyse the relationship between urbanization and schistosomiasis infection risk. We found that urbanization emerges as the second most influential factor affecting schistosomiasis infection risk, surpassing the impact of other natural factors. Our study marks a significant contribution as it is the first to suggest that urbanization may exert a more substantial influence on schistosomiasis infection risk than natural factors. Notably, urbanization has demonstrated a risk-reducing effect on schistosomiasis infection, with its impact gradually diminishing as urbanization continues to improve.

*Schistosoma japonicum* mainly occurs in rural areas with more infected water. Urbanization reduces the chance of exposure to infected water, and thereby decreases the risk of the schistosomiasis infection. While previous studies mainly focused on the impact of natural factors on the infection risk of schistosomiasis, we analysed the impact of natural and social factors on schistosomiasis infection risk in this respect concluding that urbanization is the second strongest factor of a large number of variables affecting this risk. The regression coefficient of urbanization on the schistosomiasis infection rate from 2000 to 2002 was statistically highly significant. The main reason for the negative effect on the spread of this disease by urbanization in China is probably due to the fact that the schistosomiasis patients are mainly farmers and fishermen [48]. These population groups are particularly often infected because of their daily exposure to snail-inhabited water while lacking awareness and self-protection because of low education levels [26, 48, 49]. Since the implementation of the countrywide schistosomiasis control supported by the World Bank Loan Project in the 1990s [13, 50], Anhui Province adopted measures strengthening control activities through prohibition of livestock pasture on marshlands, replacing water buffaloes with tractors and raising livestock in captivity [50]. At the same time, Anhui Province vigorously promoted urbanization that brought about intensified economic, cultural and medical levels, leading to the migration of a large number of residents from rural areas to cities and towns. More people could enjoy a better living standard, safe surrounding environment and adequate medical resources, while the agricultural mechanization had a positive influence on the prevention and control of schistosomiasis as it reduced the risk of water contact. With the improvement of urbanization and the effective implementation of control measures, the schistosomiasis infection risk in Anhui Province declined steadily. The overall effect has been remarkable and the risk of contact with water with infected snails has become much reduced since 2003.

In previous studies, social and economic data were used to calculate the urbanization level by division into single and composite indices that comprehensively considered population, economy and social life [2]. We calculated the urbanization level by DMSP-OLS and NPP-VIIRS night-time light data, observed by sensors carried by meteorological satellites, with the advantages of strong timeliness, high economy and good comprehensiveness [51–54]. Night-time light data are strongly associated with human social and economic activities thereby conveniently reflecting the urbanization level [54, 55]. This visual presentation of the urbanization level has been successfully used in urbanization-related research on air quality [53, 56], agro-ecosystem [57] and human disease, e.g., chronic kidney disease [58]. Here, we used night-time light data to analyse the influence of urbanization on schistosomiasis infection risk demonstrating the feasibility of this approach in the field of infectious diseases.

The regression results of GTWR model show that the leached soil area is the most important factor affecting the infection risk of schistosomiasis. Because of its organic matter content, slight acidity and high moisture, leached soil is very suitable for the breeding and survival of snails [17, 59] and therefore directly affects the infection risk of schistosomiasis. The next two important factors found were urbanization and distance to the nearest water source. Both of them obviously reduce the possibility of people coming into contact with infected water through activities such as swimming, fishing or agricultural production, thus strongly affecting the risk [60]. The other natural factors, e.g., average minimum temperature and precipitation, did not have a particularly significant influence on the infection risk of schistosomiasis. Our results of variable filtering suggest that we should not only consider natural factors in disease research but also social factors such as urbanization.

In this study, we compared OLS, GWR and GTWR as models of schistosomiasis infection risk. GTWR was clearly better than traditional regression methods, while the OLS model treated the parameters as constants and did not reflect the differences of risk factors in time and space. Moreover, GTWR had a better fit than traditional spatial regression models [61], while the basic GWR model only reflects the spatial differences in risk factors. Thus, the GTWR model adds temporal and spatial parameters to the regression equation, which fully reflect the temporal and spatial characteristics of the regression relationship [61]. The results fully support the usefulness of the GTWR model for analysis of the temporal and spatial non-stationarity characteristics of the data. This also verifies the accuracy of the GTWR model evaluated by scholars in the field of public health. However, it is worth mentioning that to construct a reliable regression function, the GTWR model requires an unbroken run of spatio-temporal data and explanatory variables.

Some limitations in this study deserve further discussion. Firstly, this study primarily examined the association between urbanization and schistosomiasis infection risk through statistical modelling. It did not delve into the exploration of potential mechanisms underlying this relationship. Second, the influencing factors used in the model were all abiotic. In future work, the impact of biological factors, such as farm cattle and other domestic animal, on the risk of schistosomiasis in the population can be considered to improve the credibility of the conclusions. In addition, it is impossible to collect annual vegetation cover data and soil data, therefore it is necessary to use the same data from 2000 to 2015, which is a deficiency of the research. Third, the observed temporal and spatial heterogeneity in the influence of urbanization on schistosomiasis infection risk, as identified in this study, represents a valuable area for our future research endeavors. Finally, we found that the urbanization level had a statistically significant influence on schistosomiasis from 2000 to 2002, but did not reach significance after 2003. Possibly, there may be a certain threshold for the urbanization level with respect to effect on schistosomiasis infection risk, but this threshold was not evident in our study. Therefore, exploring potential heterogeneities related to the threshold of urbanization under varying conditions should be a valuable avenue for future research.

## Conclusions

The study established a GTWR model for the analysis of the influence of urbanization on schistosomiasis infection risk at the county level in Anhui Province from 2000 to 2015. It was revealed that urbanization decreases the schistosomiasis infection risk, and the influence on schistosomiasis infection risk decreases as urbanization increases. The urbanization was only second to leached soil area and had a greater effect on schistosomiasis infection risk than most previously reported natural environmental factors. The results suggest that we should make greater efforts to spread awareness of prevention and improve urbanization to reduce people's exposure to snails and infected water in areas with a high prevalence of schistosomiasis. The study emphasizes the comprehensive consideration of the impact of natural and social factors on the infectious disease and provides a methodological basis for the quantitative risk factor analysis of infectious diseases from a spatial–temporal perspective.

### Supplementary Information

**Additional file 1.** Collinearity and significance test of independent variables. Natural factors were retained through the variable filtering.

**Additional file 2.** Accuracy comparisons. AICc values and *R*^2^ values of OLS, GWR, and GTWR models.

## Data Availability

We have a cooperation agreement with our collaborators. We can provide data if the scope of use is clear. For the software or programs related to the analysis methods in the study, we used SPSS and ArcGIS, as well as R. Most of them are interface-based operating software, which is explained in the methods section of the article. If any question, please contact the corresponding authors of the article at any time.

## Abbreviations

GTWR: Geographically and temporally weighted regression
OLS: Ordinary least square
GWR: Geographically weighted regression
AIC: Akaike information criterion
AIPD: Anhui Institute of Parasitic Disease
DMSP-OLS: Defense Meteorological Satellite Program-Operational Linescan System
NPP-VIIRS: National Polar-Orbiting Partnership’s Visible Infrared Imaging Radiometer Suite;
NASA: US National Aeronautics and Space Administration
LAADS DAAC: Level-1 and Atmosphere Archive & Distribution System-Distributed Active Archive Center
VIF: Variance inflation factor
ANLI: Average night-time light lndex
