# Supplementary material for: Influence of urbanization on schistosomiasis infection risk in Anhui Province based on sixteen year's longitudinal surveillance data: a spatio-temporal modelling study
Source: Infect Dis Poverty. 2023 Nov 29;12:108. doi: 10.1186/s40249-023-01163-3 (PMC10685489; doi:10.1186/s40249-023-01163-3)
Supplement: Supplementary file 1 — Additional file 1. Collinearity and significance test of independent variables. Natural factors were retained through the variable filtering. [file 40249_2023_1163_MOESM1_ESM.docx]

**Table S1. Collinearity and significance test of independent variables**

| **Serial number** | Influence **factor** | | **VIF** | ***P*** |
| --- | --- | --- | --- | --- |
| 1 | | Surface temperature | 2.444 | 0.000 |
| 2 | | Soil bulk density | 8.339 | 0.000 |
| 3 | | Broadleaf forest area | 4.585 | 0.026 |
| 4 | | Grassland area | 8.616 | 0.000 |
| 5 | | Leached soil area | 7.047 | 0.000 |
| 6 | | Nearest water source (dist.) | 3.898 | 0.045 |
| 7 | | Amount of precipitation | 8.163 | 0.000 |
| 8 | | Mean minimum temperature | 2.698 | 0.003 |

* VIF: Variance inflation factor
