# Supplementary material for: Influence of urbanization on schistosomiasis infection risk in Anhui Province based on sixteen year's longitudinal surveillance data: a spatio-temporal modelling study
Source: Infect Dis Poverty. 2023 Nov 29;12:108. doi: 10.1186/s40249-023-01163-3 (PMC10685489; doi:10.1186/s40249-023-01163-3)
Supplement: Supplementary file 2 — Additional file 2. Accuracy comparisons. AICc values and R2 values of OLS, GWR, and GTWR models. [file 40249_2023_1163_MOESM2_ESM.docx]

**Table S2. Accuracy comparisons**

| **Statistics** | **OLS** | **GWR** | **GTWR** |
| --- | --- | --- | --- |
| AICc | -710.841 | -722.873 | -773.885 |
| *R*2 | 0.217 | 0.217 | 0.375 |
